# The subgingival microbiome in periodontitis: from ecological dysbiosis and metabolic reprogramming to precision interventions

**DOI:** 10.3389/fmicb.2026.1906205

**Published:** 2026-08-07

**Authors:** Ping Peng, Jingpeng Cai, Linglin Zhang, Youcheng Lu, Panpan Kuang

**Affiliations:** Oral Department of Kunming Tongren Hospital, Kunming, Yunnan, China

**Keywords:** dysbiosis, metabolic reprogramming, microbiota-host interaction, oral microbiome, periodontal-systemic axis, precision intervention

## Abstract

Periodontitis is a dysbiosis-driven chronic inflammatory disease characterized by complex interactions among the subgingival microbiome, microbial metabolism, and host immune responses. Accumulating evidence indicates that disease progression is not solely determined by the enrichment of specific periodontal pathogens but is critically associated with ecological disruption and functional reprogramming of the subgingival microbial community. During the transition from periodontal health to disease, microbial metabolism shifts from carbohydrate utilization toward proteolysis and amino acid fermentation, resulting in altered production of short-chain fatty acids, polyamines, volatile sulfur compounds, hydrogen sulfide, and nitric oxide. These metabolic alterations contribute to inflammasome activation, immune dysregulation, osteoclastogenesis, and progressive periodontal tissue destruction. Beyond local pathology, periodontal microorganisms and their metabolites can disseminate through the oral–systemic axis, thereby influencing the pathogenesis of multiple systemic disorders. Recent advances in multi-omics technologies have further revealed that metabolic reprogramming represents a critical mechanistic link connecting ecological dysbiosis with host inflammatory responses. Consequently, therapeutic strategies are evolving from conventional antimicrobial approaches toward precision microbiome-based interventions, including probiotics, postbiotics, bacteriophages, predatory bacteria, metabolic modulation, and oral microbiome transplantation. In this review, we integrate current evidence on microbial ecology, metabolism, and host interactions and propose the Ecological Dysbiosis–Metabolic Reprogramming–Host Crosstalk framework. This framework highlights metabolic reprogramming as the central bridge linking microbial dysbiosis to host inflammatory damage and provides a conceptual basis for the development of precision periodontal medicine.

## 1 Introduction

Periodontitis is a chronic inflammatory disease characterized by progressive destruction of the tooth-supporting tissues and remains one of the leading causes of tooth loss worldwide (Papapanou et al., 2018). It affects approximately 20–50% of the global population, with prevalence and severity increasing with age (Kassebaum et al., 2014). According to the Global Burden of Disease study, more than 500 million individuals suffer from severe periodontitis, and its burden is expected to increase further as populations age (GBD 2017 Causes of Death Collaborators, 2018). Beyond causing local tissue destruction, periodontitis has been increasingly associated with systemic disorders, including cardiovascular disease, diabetes, gastrointestinal disorders, and neurodegenerative diseases, highlighting its importance as a component of the oral–systemic axis (Sanz et al., 2020; Preshaw et al., 2012; Fisher et al., 2008; Koziel et al., 2014; Corbella et al., 2016).

Scaling and root planing (SRP) remains the cornerstone of non-surgical periodontal therapy and effectively reduces microbial burden and inflammation (Sanz et al., 2020). However, long-term treatment outcomes are often limited by residual biofilms, microbial recolonization, and substantial interindividual variability (Harrel et al., 2022; Ramanauskaite and Machiulskiene, 2020). Although adjunctive antibiotics may improve short-term outcomes, their prolonged use can promote the enrichment of antibiotic-resistant microorganisms and antibiotic resistance genes (ARGs), further disrupting microbial homeostasis (Kang et al., 2021). These limitations suggest that eliminating pathogens alone is insufficient for achieving durable periodontal health and underscore the need for a deeper understanding of microbial ecosystem function. Advances in high-throughput sequencing and multi-omics technologies have fundamentally transformed our understanding of periodontal disease. Periodontitis is no longer viewed as an infection caused by a limited number of pathogens but rather as a complex ecological disorder arising from interactions among microbial dysbiosis, host immune dysfunction, and environmental influences. This conceptual transition is reflected in the evolution of etiological theories. The classical specific pathogen hypothesis emphasized a small group of microorganisms, particularly the “red complex” species *Porphyromonas gingivalis*, *Tannerella forsythia*, and *Treponema denticola* (Socransky et al., 1998). However, the oral cavity harbors more than 700 bacterial species together with diverse viral, fungal, and archaeal communities (Pathak et al., 2021). Increasing evidence indicates that periodontitis involves broad alterations in community structure and ecological interactions rather than the expansion of individual pathogens alone (Baker et al., 2017). Accordingly, Hajishengallis and Lamont proposed the dysbiosis paradigm, in which keystone pathogens reshape the local environment and host immunity, driving the microbial community toward a disease-associated state (Hajishengallis and Lamont, 2012). The identification of previously underappreciated community members, including TM7 (Saccharibacteria), *Candida albicans*, and bacteriophages, has further emphasized the ecological complexity of the periodontal microbiome (He et al., 2015; Chipashvili et al., 2021; Bartnicka et al., 2020).

Concurrently, periodontal research has shifted from asking “who is there” to understanding “what they are doing”. Taxonomic composition alone cannot fully explain disease activity, tissue destruction, or patient-to-patient heterogeneity. In contrast, microbial metabolic functions more directly reflect ecological status and biological activity within the periodontal niche. Emerging evidence suggests that disease-associated microbial communities undergo profound metabolic reprogramming, resulting in altered production of short-chain fatty acids, amino acid metabolites, polyamines, sulfur compounds, and other bioactive molecules that influence host immunity and tissue homeostasis. Consequently, metabolic reprogramming is increasingly recognized as a critical mechanistic link connecting microbial dysbiosis to host inflammatory damage.

**Figure 1:**
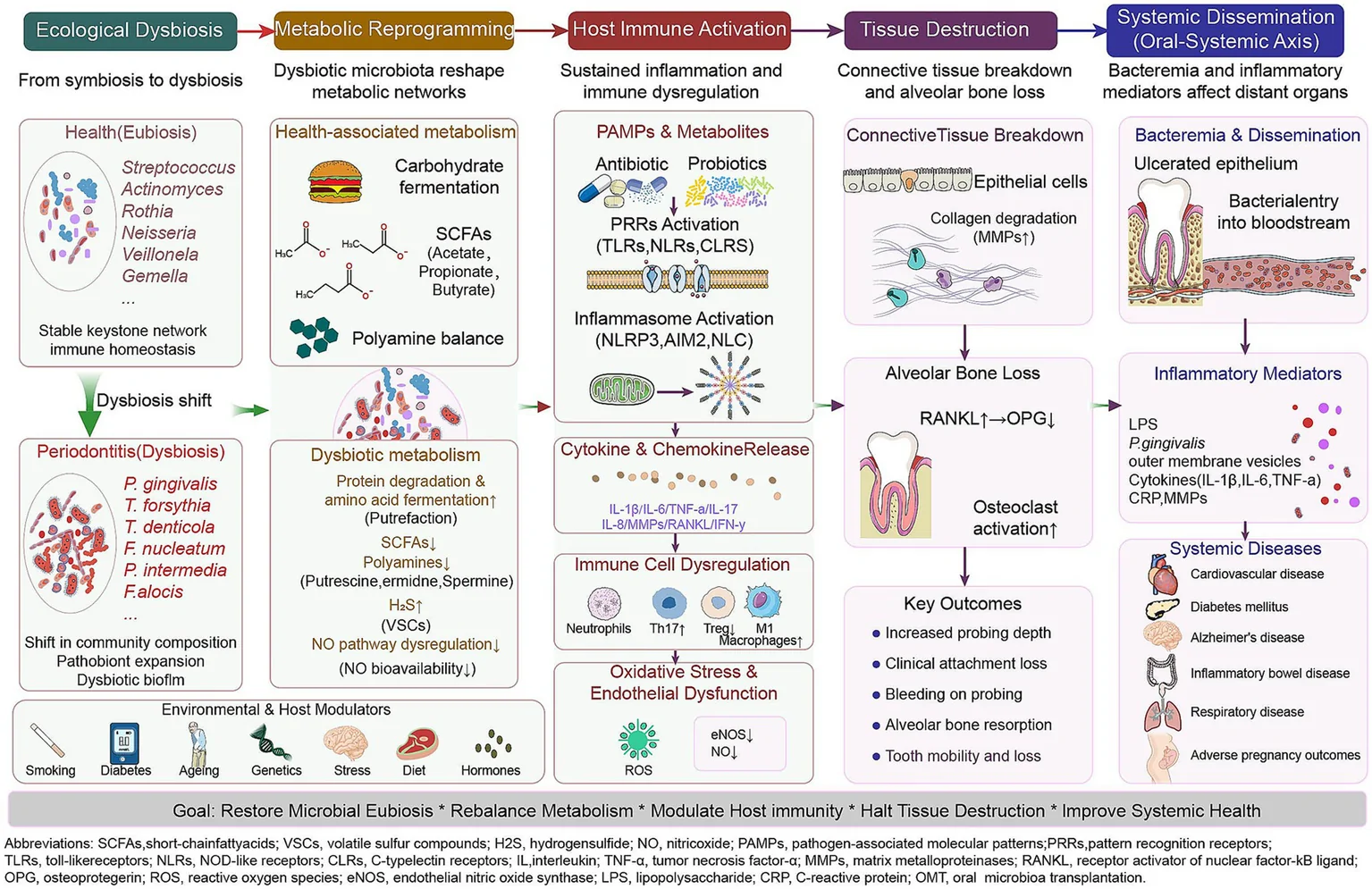
Proposed EMH framework in periodontitis. Ecological dysbiosis promotes microbial metabolic reprogramming, leading to immune activation, connective tissue degradation, alveolar bone loss, and systemic dissemination of microbial products and inflammatory mediators. Metabolic reprogramming serves as a central mechanistic bridge linking subgingival dysbiosis to periodontal destruction and systemic disease through the oral-systemic axis.

Despite significant advances in microbiome characterization and immune profiling, current studies largely investigate microbial composition, metabolism, and host responses as separate processes. A unified framework integrating these dimensions remains lacking. In particular, how ecological dysbiosis drives metabolic reprogramming and how metabolic alterations subsequently shape host immune responses and tissue destruction are still incompletely understood. To address this gap, we propose the Ecological Dysbiosis–Metabolic Reprogramming–Host Crosstalk (EMH) framework (Figure 1). This framework positions metabolic reprogramming as the central mechanistic bridge linking microbial dysbiosis to host pathology and is based on three core concepts: (i) metabolic reprogramming is the functional hallmark of periodontal dysbiosis; (ii) microbial metabolites serve as key mediators connecting microbial ecology and host responses; and (iii) metabolic modulation represents a promising target for precision microbiome-based interventions. Guided by the EMH framework, this review synthesizes current knowledge of ecological dysbiosis, metabolic reprogramming, and host–microbiome interactions in the subgingival ecosystem and discusses emerging therapeutic strategies, including probiotics, postbiotics, bacteriophages, metabolic modulation, and oral microbiome transplantation (OMT). By integrating these advances within a unified conceptual framework, we aim to provide new mechanistic insights and therapeutic opportunities for precision periodontal medicine.

## 2 Composition of the subgingival microbiota in health and disease

### 2.1 Core microbial communities associated with periodontal health

The healthy subgingival microbiota constitutes a relatively stable microbial ecosystem that exists in a state of mutualistic coexistence with the host. Data from the Human Microbiome Project and subsequent large-scale sequencing studies have revealed several characteristic features of the healthy subgingival community. At the phylum level, the microbiota is predominantly composed of Firmicutes, Actinobacteria, Bacteroidetes, Proteobacteria, and Fusobacteria. At the genus level, *Streptococcus* and *Actinomyces* are among the most abundant taxa, collectively accounting for approximately 40–60% of the total microbial population (Segata et al., 2012; Griffen et al., 2012). These Gram-positive commensals play pivotal roles in maintaining microbial homeostasis. In particular, *S. sanguinis*, *S. gordonii*, and *S. oralis* are recognized as early colonizers of the tooth surface. Beyond establishing the structural foundation of the oral biofilm, these species contribute to colonization resistance through the production of hydrogen peroxide (H₂O₂), which can suppress the growth of periodontal pathogens (Segata et al., 2012; Griffen et al., 2012; Xu et al., 2020). Another important function of the health-associated microbiota is the maintenance of the nitrate–nitrite–nitric oxide (NO₃^−^–NO₂^−^–NO) pathway. Certain commensal taxa, including species within the genera *Neisseria*, *Rothia*, and *Haemophilus*, express nitrate reductases that convert dietary nitrate into nitrite, which can subsequently be reduced to NO, a bioactive molecule with vasodilatory, anti-inflammatory, and antimicrobial properties (Rosier et al., 2020). This pathway contributes not only to local tissue perfusion and immune surveillance but also to systemic cardiovascular health.

In addition, the healthy subgingival microbiota maintains ecological stability through several complementary mechanisms. Commensal microorganisms maintain periodontal homeostasis through colonization resistance, antimicrobial activity, and environmental stabilization (Wang and Kuramitsu, 2005). Acids generated through microbial metabolism are counteracted by salivary buffering systems and ammonia produced by urease-positive bacteria, preventing excessive local acidification (Nascimento et al., 2014).

Collectively, the healthy periodontal microbiota represents a highly organized and functionally integrated microbial community that provides essential colonization resistance, supports immune homeostasis, and contributes to the maintenance of periodontal health. The key taxonomic and functional characteristics of health-associated oral microbiota are summarized in Table 1.

**Table 1:** Taxonomic composition, representative taxa and functional roles of the healthy oral microbiome.

Taxonomic level	Representative taxa	Major ecological niche	Primary function	Key mechanism	References
Phylum Firmicutes	*Streptococcus*, *Veillonella*	Dental surfaces, saliva	Carbohydrate metabolism, ecological homeostasis	Glycolysis and lactate metabolism	Dewhirst et al. (2010)
Phylum Actinobacteria	*Actinomyces*, *Rothia*	Tooth surfaces, gingival margin	Initial colonization and biofilm establishment	Adhesion proteins and extracellular matrix production	Dewhirst et al. (2010)
Phylum Proteobacteria	*Neisseria*, *Haemophilus*	Saliva, tongue dorsum	Nitrate reduction and NO production	Nitrate–nitrite–NO pathway	Rosier et al. (2020)
Phylum Bacteroidota	*Prevotella*, *Capnocytophaga*	Gingival crevice	Protein and peptide metabolism	Proteolytic activity	Kilian et al. (2016)
Phylum Fusobacteriota	*Fusobacterium nucleatum*	Dental plaque biofilm	Structural integration of multispecies communities	Coaggregation and bridging interactions	Kolenbrander et al. (2010)
Species	*S. salivarius*	Tongue dorsum	Mucosal homeostasis and immune modulation	Bacteriocin secretion	Kilian et al. (2016)
Genus	*Rothia* spp.	Saliva	Nitrate reduction	NO generation	Rosier et al. (2020)
Genus	*Actinomyces* spp.	Dental plaque	Early biofilm development	Surface adhesins	Dewhirst et al. (2010)
Genus	*Veillonella* spp.	Dental plaque	Lactate utilization	Metabolic cross-feeding	Periasamy and Kolenbrander (2010)
Fungi	*Candida albicans*	Oral mucosa	Commensal colonization and opportunistic pathogenicity	Biofilm interactions with bacteria	Montelongo-Jauregui and Lopez-Ribot (2018)
Viruses	Oral bacteriophages	Multiple oral habitats	Regulation of bacterial community structure	Host bacterial lysis and horizontal gene transfer	Baker et al. (2017)
Archaea	*Methanobrevibacter oralis*	Subgingival niche	Hydrogen scavenging	Methanogenesis	Wade (2013)
Functional Group	Health-associated microbial communities	Entire oral cavity	Colonization resistance	Competition for nutrients and adhesion sites	Kilian et al. (2016)
Functional Group	Nitrate-reducing consortium (*Neisseria*, *Rothia*)	Saliva	Cardiovascular protection	Nitrate → nitrite → NO conversion	Rosier et al. (2020)
Functional Group	Biofilm scaffold bacteria (*Fusobacterium*)	Dental plaque	Biofilm architecture stabilization	Coaggregation networks	Kolenbrander et al. (2010)

### 2.2 Periodontitis-associated microbial dysbiosis

#### 2.2.1 From the “red complex” to a more complex pathogenic network

The abundance of red complex bacteria, including *P. gingivalis*, *Tannerella forsythia*, and *T. denticola*, is markedly increased in patients with periodontitis. These organisms interact synergistically with members of the orange complex, such as *Fusobacterium nucleatum*, *Prevotella intermedia*, *Campylobacter rectus*, and *Parvimonas micra*, forming a highly organized pathogenic biofilm that contributes to disease progression (Diaz and Valm, 2020). In addition to the red complex, several opportunistic pathobionts are enriched in periodontitis. These include multiple species within the genus *Prevotella* (e.g., *P. intermedia*, *P. nigrescens*, and *P. denticola*), as well as members of the genera *Selenomonas* and *Eubacterium*, *Filifactor alocis,* and the recently recognized periodontal pathobiont *Peptoanaerobacter stomatis* (Pérez-Chaparro et al., 2014). Among them, *F. alocis* has attracted considerable attention as a fast-growing, strictly anaerobic Gram-positive rod that is strongly associated with periodontal disease. Both its prevalence and relative abundance are significantly higher in periodontitis patients than in healthy individuals, making it a potential microbial marker of disease activity (Aruni et al., 2014).

**Figure 2:**
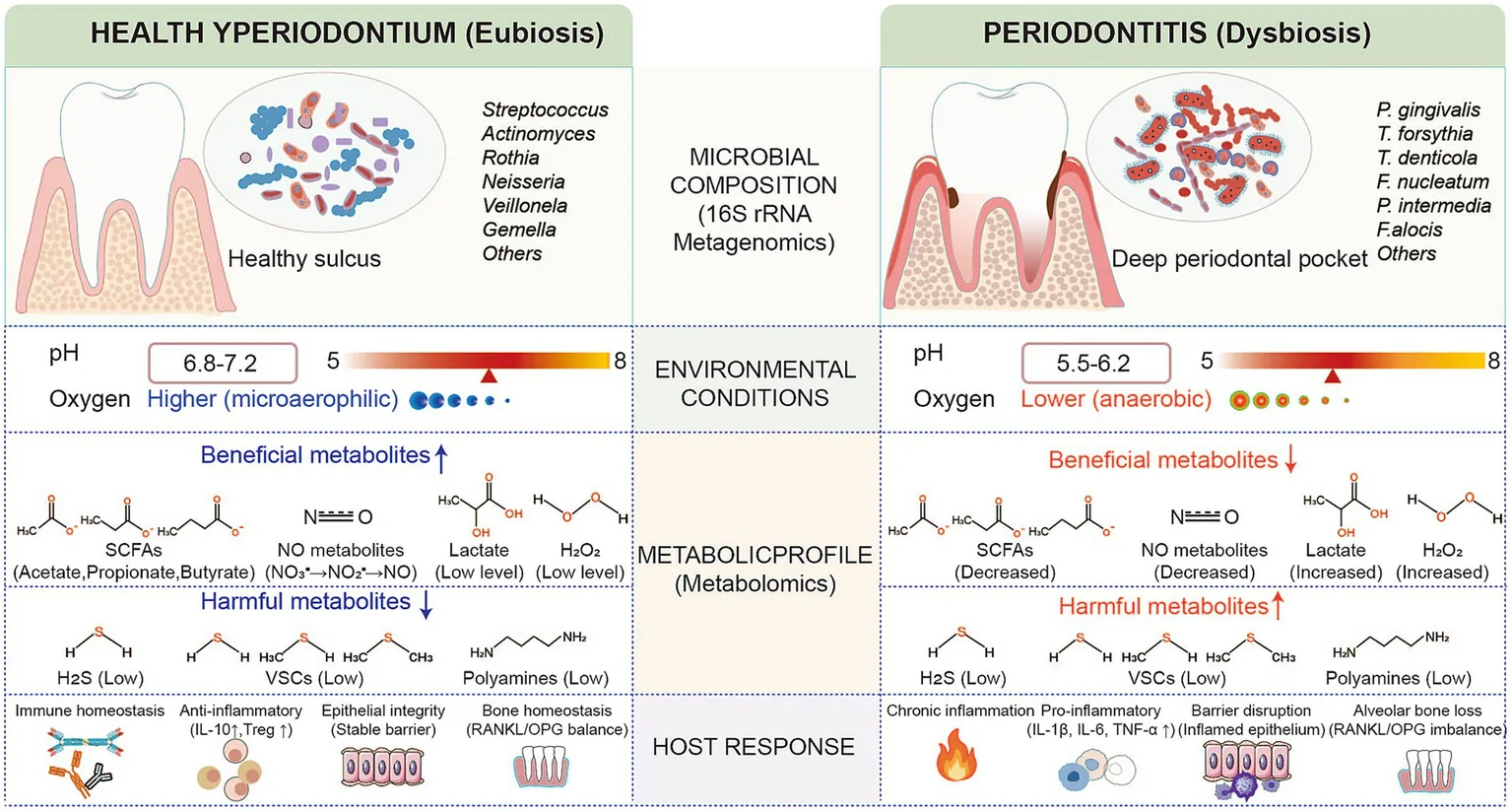
Subgingival microbial and metabolic shifts from health to periodontitis. In health (left), the subgingival microbiota is dominated by aerobes/facultative anaerobes, utilizing glycolysis and the nitrate–nitrite–NO pathway, with neutral pH and higher oxygen tension (6.8–7.2% O_2_). In periodontitis (right), dysbiosis favors obligate anaerobes (“red complex” and pathobionts), driving metabolic reprogramming toward proteolysis/amino acid fermentation. This yields toxic metabolites (H_2_S, polyamines, elevated butyrate), impairs nitrate reduction, and sustains inflammation-driven tissue destruction and bone resorption.

Importantly, periodontal dysbiosis is characterized not only by the enrichment of pathogenic taxa but also by the depletion of health-associated microorganisms. During the transition from periodontal health to disease, the subgingival microbiota frequently exhibits a reduction in *α*-diversity, accompanied by the loss of beneficial taxa, including members of the genera *Streptococcus*, *Rothia*, *Neisseria*, and *Actinomyces* (Abusleme et al., 2013). The depletion of these health-associated microorganisms weakens colonization resistance and compromises protective host functions, such as nitrate-dependent NO production, thereby further promoting microbial imbalance and disease progression (Figure 2).

#### 2.2.2 Beyond bacteria: contributions of other microbial kingdoms

The complexity of the subgingival microbiota extends far beyond bacteria alone. Increasing evidence has highlighted the potential involvement of fungi, viruses, and archaea in the pathogenesis of periodontitis. *Candida albicans* is the most prevalent fungal commensal in the human oral cavity and the primary etiological agent of oral candidiasis. Both the prevalence and abundance of *C. albicans* have been reported to be significantly elevated in patients with periodontitis (Canabarro et al., 2013). Beyond bacteriophages, herpesviruses, including Epstein–Barr virus (EBV) and human cytomegalovirus, have also been associated with severe periodontitis and may indirectly promote bacterial infection through local immunosuppressive effects (Baker et al., 2024). Archaea represent one of the least explored components of the oral microbiome. To date, all identified oral archaea belong to methanogenic lineages, among which *Methanobrevibacter oralis* is the most frequently detected species (Lepp et al., 2004). Although typically present at low abundance, both the prevalence and load of oral archaea are significantly increased in periodontitis-associated subgingival communities (Lenartova et al., 2021). Through methanogenesis, these organisms consume molecular hydrogen (H₂), thereby creating favorable metabolic conditions for acidogenic microorganisms and contributing to microbial community restructuring during disease progression (Parveen et al., 2024). Taken together, periodontal dysbiosis should be viewed as a cross-kingdom ecological disturbance involving bacteria, fungi, viruses, and archaea. This complexity extends well beyond the traditional bacteria-centered paradigm and underscores the need to investigate periodontitis from an ecosystem-wide perspective.

Collectively, available evidence indicates that periodontal dysbiosis is characterized by a bidirectional imbalance. On one hand, periodontal pathogens and opportunistic microorganisms, including members of the red complex, become enriched; on the other hand, health-associated taxa are progressively depleted. This dual shift transcends the classical specific-pathogen model and reflects a broader collapse of microbial community organization. More importantly, the loss of beneficial microorganisms results in the impairment of critical protective functions, including nitrate reduction and pH buffering, thereby establishing the functional basis for subsequent metabolic reprogramming.

## 3 Metabolic reprogramming of the subgingival microbiota and its pathogenic implications

### 3.1 Concept of metabolic reprogramming

Although considerable interindividual variation exists in the taxonomic composition of the subgingival microbiota, periodontitis-associated communities display a remarkable convergence in metabolic function. A hallmark of this transition is the shift from carbohydrate-based metabolism toward proteolysis and amino acid fermentation. Understanding the mechanisms underlying this shift and its pathogenic consequences is therefore critical for elucidating periodontal disease progression. This process, termed metabolic reprogramming, refers to the functional reorganization of microbial metabolic networks from a symbiotic state to a disease-associated pathogenic state. In periodontitis, metabolic reprogramming is characterized by a transition from glycolysis-dominated metabolism toward protein degradation and amino acid fermentation, accompanied by selective enrichment and depletion of metabolic pathways. Notably, despite substantial differences in microbial composition among patients, the metabolic capabilities of periodontitis-associated communities remain relatively conserved, suggesting that functional alterations may play a more important role in disease progression than taxonomic changes alone (Sakanaka et al., 2022). Under healthy conditions, subgingival microorganisms primarily utilize carbohydrates and generate metabolites such as lactate, acetate, and formate through glycolysis, supporting metabolic homeostasis within the oral ecosystem (Takahashi, 2015). In contrast, inflammatory tissue breakdown and increased gingival crevicular fluid during periodontitis provide abundant heme, iron, proteins, and peptides, creating conditions that favor proteolytic metabolism. As a result, microbial metabolism shifts toward protein degradation and amino acid fermentation (Takahashi et al., 2010). Metagenomic analyses further support this transition, revealing enrichment of pathways involved in pentose and glucuronate interconversions, fructose and mannose metabolism, and galactose metabolism in periodontitis-associated communities (Lu et al., 2022). These metabolic adaptations not only supply microorganisms with energy and nutrients but also generate bioactive metabolites with cytotoxic, immunomodulatory, and tissue-destructive effects. The drivers of microbial metabolic reprogramming are likely multifactorial, including inflammatory microenvironmental changes that alter substrate availability, restructuring of metabolic functions following community shifts, and rewiring of metabolic cross-feeding interactions between pathogenic and commensal microorganisms (Li et al., 2025; Suarez et al., 2026). Collectively, these findings highlight metabolic reprogramming as a central feature of periodontal dysbiosis and support microbial metabolism as a promising therapeutic target beyond conventional pathogen eradication.

### 3.2 Alterations in key metabolites and their pathogenic effects

#### 3.2.1 The shift from carbohydrate metabolism to proteolysis and amino acid fermentation

A hallmark of periodontal dysbiosis is the metabolic transition from glycolysis-dominated carbohydrate utilization to proteolytic metabolism and amino acid fermentation (Masuda et al., 2001). Major periodontal pathogens, including *P. gingivalis*, *T. denticola*, and *Tannerella forsythia*, possess limited carbohydrate-fermenting capacity and rely heavily on the catabolism of amino acids, particularly arginine, lysine, and aspartate, as primary energy sources. This metabolic shift promotes pathogen persistence, metabolic cooperation, and adaptation to the inflammatory niche.

When cocultured with *S. gordonii*, *F. nucleatum* utilizes ornithine released by the latter to produce putrescine. Likewise, coculture with *Veillonella parvula* promotes the production of cadaverine (Sakanaka et al., 2022). These polyamine metabolites exert profound effects on the biofilm life cycle of *P. gingivalis*. In particular, putrescine has been shown to accelerate the transition from planktonic growth to biofilm maturation and subsequent biofilm dispersal, thereby facilitating microbial dissemination and ecological adaptation. Collectively, *F. nucleatum* also serves as a metabolic hub facilitating cross-feeding interactions. Through the coordination of nutrient exchange and metabolite production, this network creates a favorable ecological environment for the expansion and persistence of periodontal pathogens, thereby promoting disease progression.

#### 3.2.2 The dual role of short-chain fatty acids

Short-chain fatty acids (SCFAs), particularly butyrate, propionate, and acetate, are major end products of microbial metabolism. Under physiological conditions, low concentrations of SCFAs contribute to periodontal homeostasis by modulating immune cell function through the inhibition of histone deacetylases, thereby promoting local immune tolerance (Lu et al., 2022). In periodontitis, however, SCFA concentrations, especially butyrate, can increase substantially, reaching millimolar levels within periodontal pockets. Under these conditions, their biological effects shift from protective to pathogenic. At elevated concentrations, butyrate impairs immune defense, inhibits tissue repair, and promotes inflammatory bone resorption. High levels of SCFAs can also stimulate inflammatory signaling through the activation of G protein-coupled receptors, including GPR41 and GPR43, resulting in increased production of pro-inflammatory cytokines such as IL-6 and IL-8. Furthermore, butyrate has been associated with an increased receptor activator of nuclear factor-κB ligand/osteoprotegerin ratio, thereby promoting osteoclast activation and alveolar bone resorption. Collectively, these findings suggest that SCFAs function as a double-edged sword in periodontal tissues, with their biological effects being highly dependent on concentration and local environmental context (Guan et al., 2021).

#### 3.2.3 Hydrogen sulfide and volatile sulfur compounds

Periodontal pathogens, including *P. gingivalis*, generate hydrogen sulfide (H₂S) and other volatile sulfur compounds (VSCs), such as methyl mercaptan (CH₃SH), through the degradation of sulfur-containing amino acids, including cysteine and methionine. Although VSCs are widely recognized as the principal contributors to oral malodor, their pathogenic significance extends far beyond halitosis. H₂S exerts multiple deleterious effects within the periodontal microenvironment. At the cellular level, H₂S can inhibit cytochrome c oxidase (mitochondrial complex IV), leading to mitochondrial dysfunction and excessive production of reactive oxygen species (ROS), thereby exacerbating oxidative stress (Calenic et al., 2010). H₂S also compromises epithelial barrier integrity by disrupting tight junction proteins, including occludin and claudin, resulting in increased epithelial permeability and facilitating the penetration of microorganisms and their virulence factors into underlying tissues (Chen et al., 2010). In addition, H₂S influences host immune responses by interfering with macrophage function through modulation of the NF-κB signaling pathway, thereby enhancing the ability of periodontal pathogens to evade immune clearance (Basic et al., 2019). Taken together, VSCs should not be viewed merely as by-products of microbial metabolism. Rather, they represent biologically active mediators generated during metabolic reprogramming that directly contribute to tissue destruction, barrier dysfunction, and immune dysregulation in periodontitis.

#### 3.2.4 Nitrogen metabolism and polyamine pathways

In periodontitis, the nitrate-reducing capacity of the subgingival microbiota is markedly impaired. This reduction is associated with the depletion of nitrate-reducing commensals, enhanced oxidation of NO₂^−^ back to NO₃^−^ under inflammatory conditions, and the preferential conversion of elevated NO₂^−^ to ammonia rather than NO by periodontal pathogens such as *P. gingivalis* (Bryan et al., 2022). As a consequence, reduced NO bioavailability compromises local blood perfusion, tissue repair, and antimicrobial defense, thereby creating a microenvironment favorable for pathogen overgrowth. Arginine metabolism represents a critical metabolic interface between health-associated and dysbiotic microbial communities. Commensal bacteria such as *S. gordonii* metabolize arginine through the arginine deiminase system, generating ornithine and ammonia. Ornithine can subsequently be converted into putrescine by the ornithine decarboxylase of *Fusobacterium nucleatum* (Guan et al., 2021). Notably, this metabolic pathway is significantly enriched in periodontitis-associated dental plaque. Increasing evidence suggests that polyamines, including putrescine, cadaverine, and spermidine, contribute to disease progression by promoting the maturation and dispersal of *P. gingivalis* biofilms and stimulating immune cells to release pro-inflammatory mediators. Moreover, oxidative degradation of polyamines generates reactive aldehydes that can directly damage periodontal tissues. Together, these findings underscore the central role of the arginine–ornithine–polyamine metabolic axis in the pathogenesis of periodontitis and highlight polyamine metabolism as a promising target for precision therapeutic intervention (Li et al., 2025).

### 3.3 Roles of key virulence factors

#### 3.3.1 Pathogenic mechanisms of gingipains

Gingipains are the principal virulence determinants of *P. gingivalis* and comprise two arginine-specific cysteine proteases (RgpA and RgpB) and one lysine-specific cysteine protease (Kgp). These enzymes play central roles in both tissue destruction and immune evasion. Gingipains can directly degrade immunoglobulins and complement components, including C3 and C5, thereby impairing humoral immune defenses and facilitating bacterial persistence. They can also cleave intercellular adhesion molecule-1 (ICAM-1) on epithelial cells, reducing polymorphonuclear neutrophil (PMN) recruitment and weakening innate immune surveillance (Li et al., 2025). Beyond their local immunomodulatory effects, *P. gingivalis* can disrupt vascular barrier function and promote systemic dissemination. Gingipains also directly degrade extracellular matrix proteins and activate latent matrix metalloproteinases (pro-MMPs), triggering a proteolytic cascade that amplifies connective tissue destruction and alveolar bone loss. Through these combined effects, gingipains function not only as virulence factors but also as key orchestrators of periodontal tissue breakdown. Given their central role in disease pathogenesis, gingipains have emerged as attractive targets for precision therapeutic intervention. The small-molecule inhibitor S-0636, which targets *P. gingivalis* glutaminyl cyclase (PgQC), has been shown to markedly reduce gingipain activity without exerting direct antibacterial effects. Notably, S-0636 decreased keratinocyte invasion by 76%, while preserving commensal microbial populations and avoiding the induction of antimicrobial resistance (Taudte et al., 2026). In addition, naturally occurring cysteine protease inhibitors derived from plant sources and egg white proteins have demonstrated effective suppression of gingipain activity in gingival crevicular fluid and exhibit lower toxicity than chlorhexidine, highlighting their potential as biologically compatible alternatives for periodontal therapy (Siewiński et al., 2025).

#### 3.3.2 Structural and functional heterogeneity of lipopolysaccharides

Structural variations in lipopolysaccharides (LPS), particularly in the acylation and phosphorylation status of lipid A, determine their ability to activate Toll-like receptor (TLR)-mediated signaling pathways. The LPS of *P. gingivalis* exhibits considerable structural heterogeneity (Bainbridge et al., 2002), resulting in relatively weak TLR4 agonistic activity and, under certain conditions, preferential signaling through TLR2-dependent pathways (Sun et al., 2010). Distinct A-LPS isoforms exert differential effects on TLR2 and TLR4 activation, and their distribution is strongly influenced by environmental factors such as hemin availability. In contrast, *Aggregatibacter actinomycetemcomitans* LPS (AaLPS) is a potent activator of TLR4-dependent inflammatory responses and contributes to periodontal tissue destruction through enhanced inflammatory signaling (Park et al., 2015). LPS can also enter the systemic circulation through compromised gingival epithelial barriers, leading to persistent endotoxemia that has been implicated in chronic inflammatory disorders (Pussinen et al., 2022). The expansion of Gram-negative periodontal pathogens increases local and circulating LPS levels, which are associated with endotoxemia, coronary artery disease, atherosclerosis, insulin resistance, and metabolic dysfunction (Kulis et al., 2025).

Collectively, these findings support a “triple-hit” model in which metabolic reprogramming promotes periodontal destruction through three interconnected mechanisms: (i) toxic metabolites generated by proteolytic metabolism directly damage periodontal tissues; (ii) impaired nitrate reduction decreases NO bioavailability and weakens endogenous protective functions; and (iii) virulence factors such as gingipains and structurally heterogeneous LPS amplify inflammation and tissue destruction. Importantly, metabolic reprogramming and virulence factor expression act synergistically. For example, hemin derived from inflammatory exudates not only supports the growth of *P. gingivalis* but also regulates lipid A remodeling and LPS structural variation. These observations suggest that targeting key metabolic pathways may provide greater therapeutic benefit than pathogen eradication alone, consistent with the EMH framework that identifies functional remodeling of the microbial ecosystem as a central driver of disease progression.

## 4 Microbiota–host immune crosstalk

### 4.1 Initiation and maintenance of local inflammation by microbial dysbiosis

The host immune response in periodontitis is initiated by the recognition of microbial signals, including microbe-associated molecular patterns such as LPS and lipoteichoic acid, by gingival epithelial cells (Moutsopoulos and Konkel, 2018). These microbial ligands are detected by pattern recognition receptors, particularly TLR2 and TLR4, expressed on epithelial cells, dendritic cells, and macrophages. Receptor activation subsequently triggers downstream NF-κB and mitogen-activated protein kinase (MAPK) signaling pathways, leading to the production of pro-inflammatory cytokines, including IL-1β, IL-6, and TNF-*α*, as well as a range of chemokines (Baek and Lee, 2023). Under physiological conditions, these innate immune responses are tightly regulated and largely confined to local tissues, thereby maintaining microbial control while preserving tissue homeostasis. In chronic periodontitis, however, persistent microbial stimulation drives a state of sustained and dysregulated inflammation. Rather than resolving infection, the inflammatory response becomes self-perpetuating, resulting in prolonged tissue damage and creating conditions that facilitate microbial persistence and systemic dissemination. In addition to bacterial components, fungal-derived molecules may also contribute to inflammatory activation.

The release of cytokines and chemokines further promotes inflammatory cell recruitment by inducing the expression of endothelial adhesion molecules, including intercellular adhesion molecule-1 (ICAM-1), vascular cell adhesion molecule-1 (VCAM-1), and E-selectin. These changes facilitate the migration of neutrophils, monocytes, and macrophages into periodontal lesions (Hajishengallis, 2014). Concurrently, activated fibroblasts increase the production of MMPs, leading to collagen degradation, while osteoclastogenesis is stimulated, resulting in progressive alveolar bone resorption. Together, these events establish a self-reinforcing inflammatory loop that drives ongoing periodontal tissue destruction. Beyond local tissue effects, chronic periodontal inflammation may exert systemic vascular consequences.

### 4.2 The vicious cycle of inflammatory dysbiosis

Inflammation induced by microbial dysbiosis actively reshapes the periodontal microenvironment, creating a self-perpetuating cycle of inflammatory dysbiosis. By increasing nutrient availability and altering local physicochemical conditions, inflammation favors the expansion of anaerobic proteolytic pathogens. During the respiratory burst, activated neutrophils generate large amounts of ROS, consuming oxygen and promoting a more anaerobic environment that supports the growth of pathogens associated with periodontal lesions (Loesche et al., 1983). Persistent exposure to inflammatory mediators and microbial metabolites can further impair neutrophil function and induce lymphocyte apoptosis, contributing to localized immune dysfunction and worsening microbial dysbiosis.

Pyroptosis has emerged as an important mechanism of periodontal tissue destruction. Activation of the NLRP3 inflammasome promotes the release of IL-1β and IL-18 and induces gasdermin D-mediated inflammatory cell death, thereby amplifying inflammation and alveolar bone resorption (Xu et al., 2022). Consistently, gingival tissues from periodontitis patients exhibit elevated expression of NLRP3, caspase-1, and gasdermin D, which correlates with disease severity (Sordi et al., 2021). In addition, both *P. gingivalis* and *F. nucleatum* can activate the AIM2 inflammasome, indicating that multiple inflammasome pathways contribute to periodontal immunopathology (Wang et al., 2020). Dysregulated host responses may also contribute to systemic inflammatory complications. *P. gingivalis* infection can induce cross-reactive antibodies against heat shock protein 60 (HSP60), potentially promoting atherosclerosis (Moutsopoulos and Konkel, 2018; Moutsopoulos and Konkel, 2018). It can also drive macrophage polarization toward a pro-inflammatory M1 phenotype while facilitating immune evasion through programmed death-ligand 1 upregulation and suppression of CD8^+^ T-cell function (Bui et al., 2019). Within the EMH framework, these interactions highlight the reciprocal reinforcement among microbial dysbiosis, metabolic adaptation, and host immune dysfunction that drives chronic inflammation and tissue destruction.

### 4.3 Pathological mechanisms of the oral–systemic axis

#### 4.3.1 Bacteremia and direct microbial translocation

**Figure 3:**
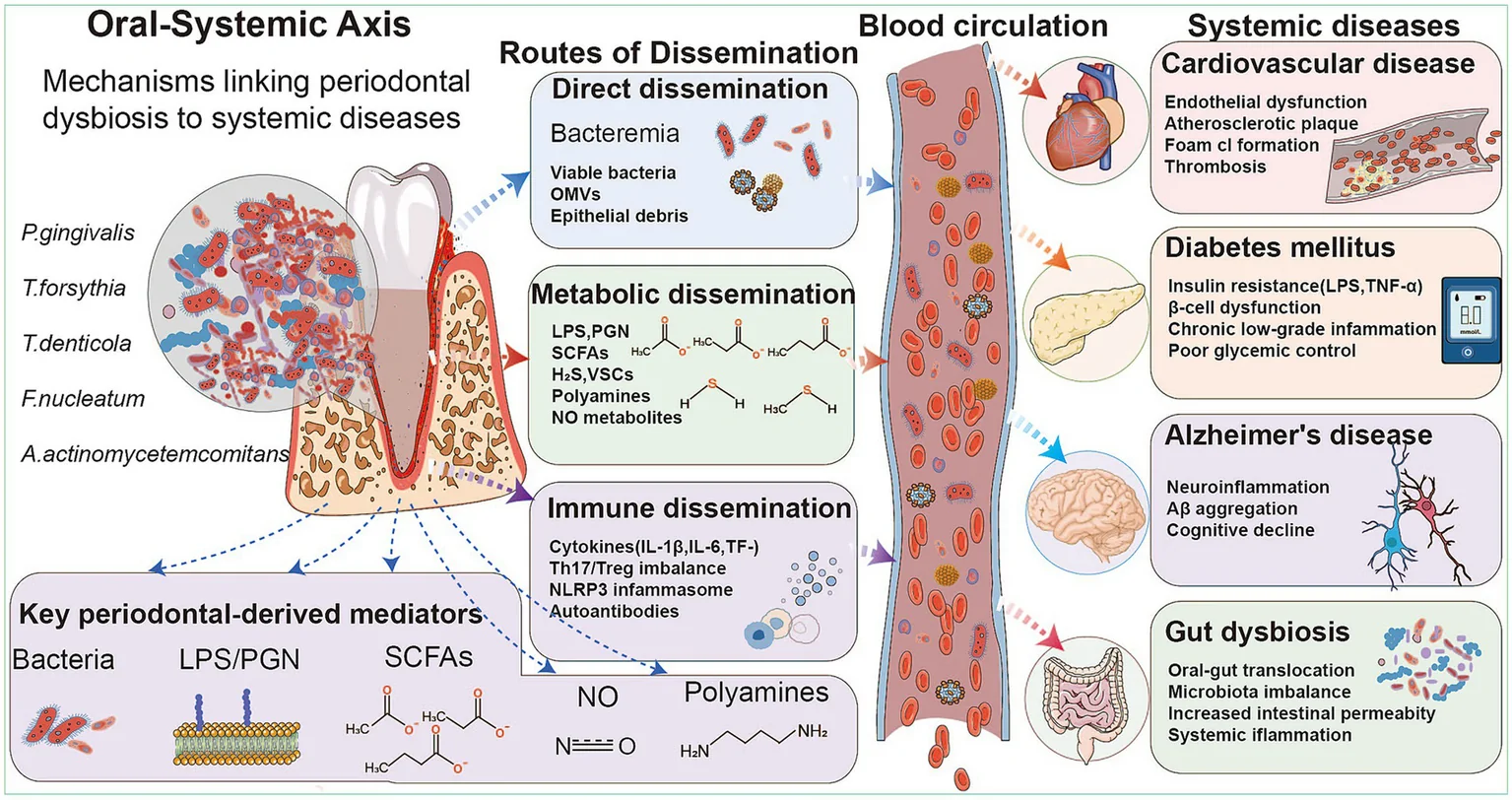
Pathways of the oral–systemic axis. Periodontal pathogens and their metabolites disseminate via direct (bacteremia, outer membrane vesicles (OMVs)), immune (cytokines, NLRP3, Th17/Treg), and metabolic (LPS, SCFAs, H_2_S, polyamines) routes. These mechanisms drive systemic diseases including cardiovascular disease, diabetes mellitus, AD, and gut dysbiosis.

In periodontitis, disruption of the gingival epithelial barrier and the formation of highly vascularized granulation tissue facilitate microbial entry into the systemic circulation (Figure 3). Once disseminated, periodontal pathogens such as *P. gingivalis*, *A. actinomycetemcomitans*, and *Fusobacterium nucleatum* can colonize distant tissues, including atherosclerotic plaques, cardiac valves, and the brain (Chhibber-Goel et al., 2016). DNA from red-complex pathogens, and in some cases viable bacteria, has been detected in atherosclerotic lesions, while experimental studies show that intravenous administration of *P. gingivalis* accelerates plaque development in ApoE-deficient mice (Lalla et al., 2003). The resulting bacteremia and endotoxemia contribute to vascular inflammation and endothelial dysfunction, key events in atherosclerosis development (Gualtero et al., 2023; Angjelova et al., 2024). Mechanistically, *P. gingivalis* can induce endothelial cell apoptosis through JAK/STAT signaling, thereby promoting atherosclerotic progression (Zhang et al., 2025). Evidence of microbial dissemination has also been reported in neurodegenerative diseases. DNA from *P. gingivalis* and gingipain antigens has been identified in the brains of patients with Alzheimer’s disease (AD) (Poole et al., 2013). Emerging evidence suggests that *P. gingivalis* may traverse the blood–brain barrier via circulating monocytes, contributing to *β*-amyloid accumulation, tau hyperphosphorylation, and neuroinflammation (Dominy et al., 2019; Mohammed et al., 2026). In addition, *P. gingivalis* LPS can activate microglia and promote pro-inflammatory responses associated with neuronal injury and AD-related neuropathology (Magnusson et al., 2024).

Collectively, these findings indicate that periodontal pathogens are not restricted to the oral cavity but can disseminate to distant organs through bacteremia and microbial translocation, contributing to systemic inflammatory and degenerative diseases. Within the EMH framework, microbial dissemination represents an extension of local ecological dysbiosis and host–microbe interactions beyond the periodontal niche, linking chronic oral infection to systemic pathology.

#### 4.3.2 Gut translocation and the oral–gut axis

Oral microorganisms are continuously delivered to the gastrointestinal tract through swallowing. Under conditions of reduced gastric acidity, impaired intestinal barrier function, or gut dysbiosis, oral microbes may colonize the intestine and contribute to disease development (Arimatsu et al., 2014). Experimental studies have shown that oral administration of *P. gingivalis* enables its colonization of the intestinal mucosa, where it disrupts epithelial barrier integrity by reducing the expression of occludin and zonula occludens-1 (ZO-1), increases intestinal permeability, promotes Th17-cell expansion, and exacerbates colitis (Nakajima et al., 2015). In addition, ARGs harbored by the oral microbiota can be transferred to gut microbial communities through horizontal gene transfer, potentially aggravating antimicrobial resistance in the intestinal ecosystem (Roberts and Kreth, 2014). Accumulating evidence indicates that oral pathogens can perturb intestinal homeostasis through multiple mechanisms, including direct microbial translocation, metabolite-mediated signaling pathways such as trimethylamine N-oxide (TMAO) and SCFAs, and immune crosstalk involving Th17 responses (Gan et al., 2026). Periodontitis induced by *P. gingivalis* has been shown to reduce the abundance of SCFA-producing gut bacteria and impair intestinal barrier function through disruption of autophagy-related pathways, particularly the ATG5–LC3 signaling axis. Restoration of autophagic activity improves barrier integrity and enhances SCFA absorption, highlighting a mechanistic link between periodontal infection and intestinal dysfunction (Sun et al., 2024).

#### 4.3.3 Immune-mediated mechanisms

Microbial components and metabolites derived from the oral microbiota, including LPS, CpG DNA, and outer membrane vesicles (OMVs), can enter the systemic circulation and trigger inflammatory responses (Cecoro et al., 2020). Patients with chronic periodontitis exhibit elevated serum levels of LPS, LPS-binding protein (LBP), soluble CD14 (sCD14), and inflammatory biomarkers such as C-reactive protein (CRP), IL-6, and TNF-*α*, which correlate positively with disease severity (Paraskevas et al., 2008). Systemic inflammatory signaling further links periodontitis with chronic diseases. Hyperglycemia-induced endothelial dysfunction can exacerbate periodontal inflammation through suppression of the PI3K/Akt/FoxO1 pathway and activation of RAGE/NF-κB and TLR2/TLR4/NF-κB signaling (Zeze et al., 2023; Tian et al., 2024). In addition, periodontal pathogens and OMVs may promote chronic inflammation and immune evasion through TLR4-dependent pathways, with their activity influenced by the structural characteristics of lipid A (Alaei et al., 2025). A recent systematic review showed that effective endodontic treatment significantly reduces circulating levels of high-sensitivity CRP and IL-1β, highlighting the contribution of oral inflammation to systemic inflammatory burden (Jakovljevic et al., 2025). Chronic periodontitis is also increasingly recognized as being bidirectionally associated with chronic kidney disease, in part through LPS-mediated activation of TLR2/TLR4 and NF-κB signaling, which promotes persistent inflammation and oxidative stress (Martínez Nieto et al., 2024).

Based on these findings, we propose an “inflammation–metabolism–microbiota” vicious cycle model within the EMH framework. This model highlights three potential intervention points: (i) limiting inflammation-derived nutrients that support pathogen growth; (ii) targeting pro-inflammatory pathways activated by microbial metabolites such as SCFAs and H₂S; and (iii) restoring endogenous host defense mechanisms, particularly the nitrate–nitrite–NO pathway.

## 5 Precision interventions: from broad-spectrum eradication to ecological modulation

### 5.1 Limitations of current therapeutic approaches

Scaling and root planing (SRP) remains the cornerstone of non-surgical periodontal therapy and is effective in removing the majority of subgingival biofilms and calculus. However, its limitations have become increasingly apparent. Although SRP remains the cornerstone of periodontal therapy, its efficacy is often limited in high-risk populations (Heitz-Mayfield and Lang, 2013). More importantly, SRP does not fully restore a health-associated microbial ecosystem. Long-term ecological stability remains difficult to achieve (Schwarzberg et al., 2014). Considerable interindividual variability in treatment outcomes has also been reported, and underscoring the need for personalized treatment strategies (Li et al., 2025). Broad-spectrum antimicrobial agents, such as chlorhexidine, exhibit potent antibacterial activity but lack microbial selectivity. Consequently, they may indiscriminately eliminate beneficial commensal species and increase the risk of microbial dysbiosis (Brookes et al., 2021; Angjelova et al., 2025). Moreover, depletion of nitrate-reducing bacteria induced by chlorhexidine may adversely affect both oral and systemic health through disruption of the nitrate–nitrite–NO pathway (Boulares et al., 2025). Systemic or locally delivered antibiotics can provide additional clinical benefits when used as adjuncts to mechanical therapy; however, the emergence and dissemination of antibiotic-resistant strains remain major concerns (Rams et al., 2014). Furthermore, some reported therapeutic benefits may have been overestimated, and the long-term advantages of adjunctive antibiotic therapy remain controversial (Gunsolley et al., 2022). Taken together, current treatment strategies that primarily focus on microbial eradication are unable to fully restore ecological homeostasis or re-establish functional microbial networks.

### 5.2 Targeted strategies for restoring microbial homeostasis

#### 5.2.1 Probiotics and postbiotics

**Figure 4:**
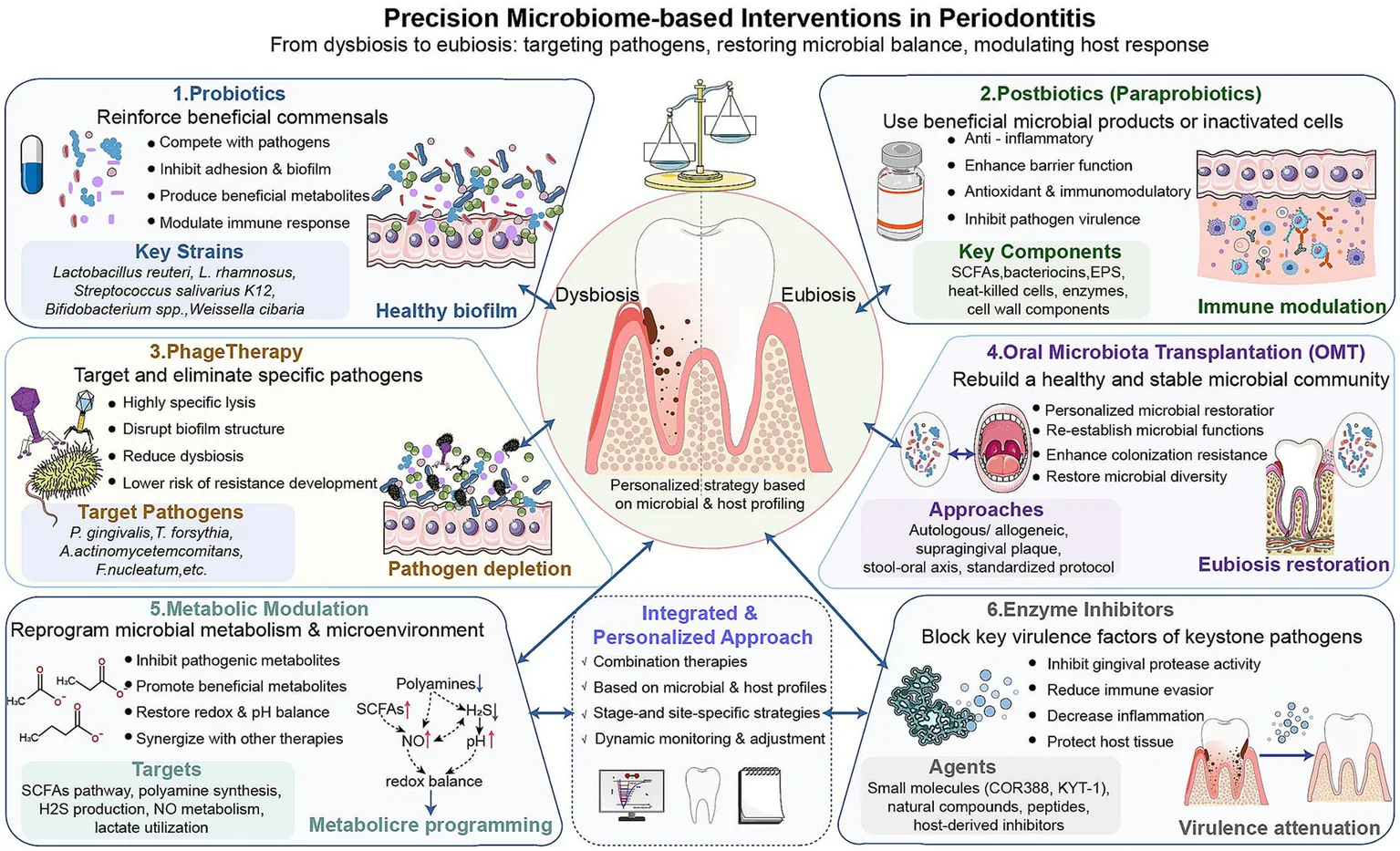
Schematic overview of emerging ecological and precision therapeutic strategies for periodontitis. (1) Probiotics - live beneficial bacteria that restore microbial homeostasis via competitive exclusion and immunomodulation; (2) Postbiotics (paraprobiotics) - inactivated microbial cells or their bioactive components (SCFAs, bacteriocins, exopolysaccharides, enzymes, cell wall components) that provide anti-inflammatory, barrier-enhancing, antioxidant, immunomodulatory, and anti-virulence effects; (3) Phage therapy - bacteriophages that specifically lyse periodontal pathogens; (4) Predatory bacteria (e.g., *Bdellovibrio bacteriovorus*) - that selectively prey on Gram-negative pathogens; (5) Metabolic modulators/prebiotics (e.g., arginine, nitrate) - that reshape the metabolic environment; (6) OMT for complete community reconstruction. The right panel also includes critical translational considerations such as delivery systems, formulation stability, safety profiles, and regulatory pathways.

Probiotics, defined as live microorganisms that confer health benefits when administered in adequate amounts, have emerged as promising adjuncts in periodontal therapy (Figure 4). Among the available candidates, Lactobacillus species have been most extensively studied, with benefits attributed to pathogen suppression and immune modulation (Twetman and Keller, 2012). Multiple meta-analyses consistently demonstrate improvements in clinical periodontal parameters following probiotic supplementation (Martin-Cabezas et al., 2016; Benavides-Reyes et al., 2025). Probiotics have also shown potential in managing halitosis, as supplementation with *Weissella cibaria* CMU significantly reduced volatile sulfur compounds and periodontal pathogens (Lee et al., 2026). Postbiotics, defined as preparations of inanimate microorganisms and/or their components that confer health benefits to the host (Salminen et al., 2021), have gained increasing attention as an alternative to live probiotics. Compared with probiotics, postbiotics offer greater stability, improved safety, and no risk of horizontal transfer of antimicrobial resistance genes (Wegh et al., 2019). *In vitro* studies have shown that postbiotic preparations derived from *L. plantarum* and *L. rhamnosus* inhibit *P. gingivalis* biofilm formation and suppress gingipain expression (Kõll-Klais et al., 2005). Despite these promising findings, challenges remain regarding long-term efficacy, dosage standardization, colonization stability, and regulatory consistency (Liu et al., 2026; Abbinante et al., 2026).

Several barriers continue to limit the routine incorporation of probiotics and postbiotics into evidence-based periodontal care. Most clinical studies have follow-up periods of less than six months, restricting assessment of long-term efficacy and safety. In addition, probiotic persistence in the oral cavity is constrained by salivary clearance and enzymatic degradation, emphasizing the need for improved delivery systems (Zidar et al., 2021). For postbiotics, optimal dosage, administration frequency, and formulation standardization remain unresolved. Variability in product quality and clinical validation further hinders their widespread adoption as standardized periodontal interventions.

#### 5.2.2 Phage therapy

Bacteriophages are viruses that specifically infect bacteria and possess a high degree of host specificity, making them attractive candidates for selectively eliminating periodontal pathogens while preserving resident commensal microbiota (Dalmasso et al., 2015). Lytic phages targeting major periodontal pathogens, including *P. gingivalis*, *A. actinomycetemcomitans*, and *Fusobacterium nucleatum*, have been successfully isolated. However, evidence supporting phage therapy against *P. gingivalis* remains largely limited to *in vitro* studies (Dalmasso et al., 2015). Compared with conventional antimicrobial approaches, phage therapy offers several advantages, including high target specificity, the ability to penetrate and disrupt biofilms, and self-amplification through replication within susceptible bacterial hosts (Jalili et al., 2025). In addition, phage-derived endolysins identified from human oral virome databases exhibit potent antibacterial activity against *P. gingivalis*, highlighting their potential as novel therapeutic agents (Xu et al., 2025).

Despite these promising findings, phage therapy remains at the preclinical stage and faces several challenges. Phage formulations may exhibit limited stability and are susceptible to enzymatic or proteolytic degradation. Effective delivery within complex oral biofilms also remains difficult, and the emergence of bacterial resistance may reduce long-term efficacy (Shlezinger et al., 2017). Furthermore, the current evidence base is limited by a lack of well-designed animal studies and clinical trials (Kabwe et al., 2025). Regulatory challenges persist, as no phage-based product for periodontal therapy has yet received marketing approval and standardized frameworks for evaluation, quality control, and clinical authorization remain underdeveloped (König, 2025).

#### 5.2.3 Predatory bacteria

Predatory bacteria are microorganisms capable of invading, killing, and consuming Gram-negative bacteria. Among them, *Bdellovibrio bacteriovorus* is the best-characterized species (Dashiff and Kadouri, 2011). *In vitro* studies have demonstrated that *B. bacteriovorus* HD100 can effectively lyse major periodontal pathogens, including *P. gingivalis*, *A. actinomycetemcomitans*, and *F. nucleatum* (Van Essche et al., 2009). Within multispecies biofilms, it preferentially targets Gram-negative pathogens while exerting minimal effects on Gram-positive commensals, thereby preserving key components of the indigenous oral microbiota (Loozen et al., 2015). Animal studies further support its therapeutic potential. Local administration of *B. bacteriovorus* HD100 significantly reduced alveolar bone loss and modulated host immune responses in experimental periodontitis models (Silva et al., 2023). These findings suggest that predatory bacteria may provide a promising strategy for selectively eliminating Gram-negative anaerobic pathogens associated with periodontal disease (Salah et al., 2025).

Several characteristics make predatory bacteria attractive candidates for microbiome-based interventions, including a low likelihood of inducing antimicrobial resistance and an inherent ability to penetrate and disrupt biofilms. However, substantial challenges remain. Predatory bacteria are highly sensitive to environmental conditions, their interactions with the host immune system remain incompletely understood, and comprehensive safety evaluations are lacking (Patini et al., 2019). In addition, current evidence is largely limited to animal studies, while standardized approaches for production, storage, quality control, and regulatory approval have yet to be established, hindering clinical translation.

#### 5.2.4 Metabolic modulation and prebiotics

Metabolic modulation aims to reshape the biochemical environment of the oral microbiome, thereby promoting health-associated microorganisms while suppressing pathogenic communities (Figure 4). Arginine supplementation enhances health-associated metabolism, increases local pH, and improves periodontal parameters. Clinical studies have shown that arginine-containing toothpastes and gels increase the abundance of beneficial oral taxa while reducing bleeding on probing and plaque accumulation (Koopman et al., 2017). Nitrate supplementation represents another promising strategy by enriching nitrate-reducing bacterial communities and reducing inflammation. Short-term use of nitrate-containing mouth rinses has been shown to increase nitrate-reducing bacteria, suppress periodontal pathogens, and improve gingival inflammatory indices (Jockel-Schneider et al., 2016). Prebiotics are selectively utilized by beneficial microorganisms and can stimulate the growth of health-associated oral taxa. Synbiotics, which combine probiotics with prebiotic substrates, are designed to enhance microbial colonization and functionality. Owing to their complementary mechanisms, synbiotics are increasingly recognized as a promising approach for oral microbiome modulation and periodontal health management (Li et al., 2025).

Despite these encouraging findings, several challenges remain. Potential systemic effects of nitrate–NO modulation, particularly on cardiovascular physiology, require further evaluation. In addition, oral prebiotic research remains limited, and most available evidence is derived from *in vitro* studies or short-term clinical trials. Consequently, further mechanistic investigations and well-designed clinical studies are needed before metabolic modulation strategies can be routinely incorporated into periodontal therapy.

#### 5.2.5 Oral microbiome transplantation

Oral microbiome transplantation (OMT) involves transferring an entire microbial community from a healthy donor to a recipient with the aim of restoring microbial homeostasis and re-establishing a balanced oral ecosystem (Figure 4). Recent studies provide preliminary support for this concept. From the perspective of the oral–gut axis, transplantation of microbiota from periodontally healthy donors has been shown to enrich beneficial microorganisms, improve barrier function, reduce systemic inflammation, and alleviate periodontal disease in experimental models, providing proof-of-concept evidence for microbiome transplantation as a strategy for restoring microbial balance (Zhang et al., 2025). Despite its theoretical appeal, OMT remains at an early stage of development and faces substantial scientific and translational challenges. Standardized donor-selection criteria have not been established, and key issues regarding engraftment efficiency, ecological compatibility, long-term stability, and safety remain unresolved. In addition, the potential transfer of antimicrobial resistance genes, opportunistic pathogens, or other undesirable microbial traits requires careful evaluation. Technical limitations related to microbiome collection, processing, preservation, delivery, and quality control further restrict clinical implementation.

Nevertheless, OMT represents a paradigm shift from microbiome modulation to microbiome reconstruction. By restoring a complete and functional microbial ecosystem rather than targeting individual pathogens, it may offer a promising therapeutic option for refractory periodontitis. However, its safety, efficacy, and long-term stability must be validated in well-designed preclinical and clinical studies before routine clinical application.

### 5.3 Prospects for personalized therapy

Increasing evidence indicates that microbial dysbiosis in periodontitis is highly heterogeneous across individuals, with substantial variation in microbial composition, functional potential, host immune responses, and disease progression patterns. Such heterogeneity has important implications for diagnosis, prognosis, and therapeutic decision-making. Among emerging microbial biomarkers, *Filifactor alocis* has shown potential for distinguishing severe forms of periodontitis and may aid disease stratification (Faisal and Ali, 2025). Functional analyses further underscore the complexity of periodontal dysbiosis. Multi-omics studies have revealed substantial alterations in microbial functional pathways associated with inflammation and metabolism. Metabolomic analyses further indicate profound shifts in metabolite profiles associated with disease progression (Baima et al., 2025). These findings support the concept that distinct microbial and metabolic signatures underlie different disease phenotypes and may require individualized therapeutic strategies.

Against this background, the next generation of precision periodontal medicine is expected to rely on the integration of multidimensional patient-specific data, including microbial community profiles, metabolomic signatures, immune phenotypes, and genomic risk scores, to guide individualized therapeutic strategies (Joshi and Sharma, 2026). Personalized interventions may be tailored according to the dominant pathogenic mechanisms identified in each patient. Therapeutic strategies may be tailored according to the dominant microbial, metabolic, and immunological characteristics of individual patients. Integration of multi-omics data with machine learning may improve disease stratification and treatment selection. As precision-based approaches continue to evolve, comparative evaluation of available microbiome-targeted interventions will become increasingly important. A summary of these key dimensions is presented in Table 2.

**Table 2:** Comparison of emerging microbiome-based interventions for periodontal disease.

Intervention strategy	Target spectrum	Highest level of evidence	Major risks and limitations	Regulatory status
Probiotics/Postbiotics	Moderate to broad (strain- and species-dependent)	Clinical trials and meta-analyses	Extremely low risk of infection, uncertain long-term colonization efficiency	Regulated primarily as foods or dietary supplements rather than pharmaceutical products
Phage therapy	Narrow (species- or strain-specific)	In vitro and ex vivo biofilm studies	Emergence of phage-resistant bacteria, limited formulation stability, low delivery efficiency within biofilms	Individualized use through *magistral formula* frameworks is permitted in certain countries, such as Belgium
Predatory bacteria	Broad (Gram-negative bacteria)	Animal studies	Sensitivity to oxygen, unknown long-term safety	No established classification system or regulatory framework
Metabolic modulation/prebiotics	Broad (ecological niche modulation)	Short-term clinical trials	Potential systemic effects, uncertain long-term impact on microbial diversity	May be marketed as food ingredients or oral care
OMT	Very broad (whole microbial community)	Proof-of-concept studies (no human clinical evidence)	Risk of infection, transmission of antimicrobial resistance genes, failure of microbial engraftment	No dedicated regulatory framework; substantial ethical and safety concerns remain

Target spectrum classified as “narrow” refers to interventions directed against a single pathogen or a limited number of bacterial species, whereas “broad” indicates effects on multiple microbial taxa or the oral ecological niche as a whole. The highest level of evidence was determined based on publicly available literature up to May 2026. Information regarding the regulatory status of phage therapy was derived from the report of the Transatlantic Taskforce on Antimicrobial Resistance (TATFAR) and related multinational working groups (Moon et al., 2025), as well as a recent review published in ScienceDirect (König, 2025). Barriers to the clinical translation and implementation of probiotic-based therapies were summarized according to the review published in Expert Opinion on Drug Delivery (Zidar et al., 2021).

## 6 Conclusions and future perspectives

In this review, we propose the EMH framework, which positions metabolic reprogramming at the center of the transition from microbial dysbiosis to host tissue damage. This framework advances three key propositions: (1) metabolic reprogramming represents the common functional consequence of microbial compositional shifts and ecological remodeling; (2) microbial metabolites serve as the principal signaling mediators of microbiota–host interactions; and (3) targeting metabolic reprogramming provides a promising avenue for precision intervention. By moving beyond traditional pathogen-centric and dysbiosis-based models, the EMH framework offers a testable conceptual basis for understanding periodontitis pathogenesis and developing next-generation therapeutic strategies. Accumulating evidence suggests that metabolic remodeling drives the transition from ecological dysbiosis to chronic inflammation and tissue destruction, supporting its central role in periodontitis pathogenesis. Within the EMH framework, metabolic reprogramming acts as a functional amplifier that translates microbial dysbiosis into host tissue injury.

Building upon this framework, several research priorities warrant particular attention in the coming years.

(1) Longitudinal studies. Longitudinal studies are needed to identify early biomarkers and ecological tipping points during disease progression.
(2) Integrated multi-omics analyses. Comprehensive integration of metagenomic, metatranscriptomic, metaproteomic, and metabolomic datasets will be essential for deciphering microbiota–host interaction networks. Integrated multi-omics analyses will be essential for deciphering microbiota–host interaction networks and identifying actionable therapeutic targets (Loozen et al., 2015; Loozen et al., 2015).
(3) Clinical translation. Large-scale randomized controlled trials are needed to systematically evaluate the efficacy and safety of emerging microbiome-based interventions, including probiotics and postbiotics, bacteriophages, predatory bacteria, and ecological modulation strategies.
(4) Discovery of novel therapeutic targets. Future efforts should focus on key regulatory nodes involved in metabolic reprogramming. The development of highly selective small-molecule inhibitors targeting these pathways may offer new opportunities for disease control. In parallel, advances in synthetic biology and genome engineering may enable the development of next-generation probiotics with enhanced antimicrobial or metabolic regulatory capabilities (Pacheco-Yanes et al., 2023).
(5) Oral–systemic integrative medicine. An important area of future investigation is whether microbiome-targeted periodontal interventions can simultaneously improve systemic inflammatory markers and clinical outcomes in patients with comorbid conditions (Kapila, 2021). Future studies should determine whether microbiome-targeted periodontal therapies can improve both oral and systemic health outcomes (Kapila, 2021).

In conclusion, viewing periodontitis through the lens of ecological dysbiosis and metabolic reprogramming provides new insights into disease pathogenesis and opens avenues for the development of transformative therapeutic approaches. Advances in multi-omics and precision medicine will facilitate the identification of actionable microbiota–host interaction networks. Ultimately, these advances may shift periodontal therapy from pathogen elimination toward ecosystem restoration and microbiome-guided precision care.
